# Effects of weather variation on waterfowl migration: Lessons from a continental‐scale generalizable avian movement and energetics model

**DOI:** 10.1002/ece3.8617

**Published:** 2022-02-17

**Authors:** Kevin J. Aagaard, Eric V. Lonsdorf, Wayne E. Thogmartin

**Affiliations:** ^1^ Colorado Parks and Wildlife Fort Collins Colorado USA; ^2^ Institute on the Environment University of Minnesota St. Paul Minnesota USA; ^3^ U.S. Geological Survey Upper Midwest Environmental Sciences Center La Crosse Wisconsin USA

**Keywords:** avian, energetics, global climate change, migration, predictive modeling, waterfowl

## Abstract

We developed a continental energetics‐based model of daily mallard (*Anas platyrhynchos*) movement during the non‐breeding period (September to May) to predict year‐specific migration and overwinter occurrence. The model approximates movements and stopovers as functions of metabolism and weather, in terms of temperature and frozen precipitation (i.e., snow). The model is a Markov process operating at the population level and is parameterized through a review of literature. We applied the model to 62 years of daily weather data for the non‐breeding period. The average proportion of available habitat decreased as weather severity increased, with mortality decreasing as the proportion of available habitat increased. The most commonly used locations during the course of the non‐breeding period were generally consistent across years, with the most inter‐annual variation present in the overwintering area. Our model revealed that the distribution of mallards on the landscape changed more dramatically when the variation in daily available habitat was greater. The main routes for avian migration in North America were predicted by our simulations: the Atlantic, Mississippi, Central, and Pacific flyways. Our model predicted an average of 77.4% survivorship for the non‐breeding period across all years (range = 76.4%–78.4%), with lowest survivorship during autumn (90.5 ± 1.4%), intermediate survivorship in winter (91.8 ± 0.7%), and greatest survivorship in spring (93.6 ± 1.1%). We provide the parameters necessary for exploration within and among other taxa to leverage the generalizability of this migration model to a broader expanse of bird species, and across a range of climate change and land use/land cover change scenarios.

## Graphical abstract

We present a model of waterfowl movement in North America during the non‐breeding period of their annual cycle. The model is based on the energetic relationships among flight dynamics and environmental conditions. We found levels of mortality commensurate with past studies (~77% across the non‐breeding period) and that habitat available for waterfowl use (based on a weather severity metric) has increased from 1957 to the present.

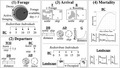

## 1 INTRODUCTION

Migratory behavior of populations varies within an avian species as well as among individuals within a population (Eichhorn et al., 2009; Newton, 2006; Newton & Brockie, 2008; Stanley et al., 2012). This differential migratory behavior is influenced by environmental change operating over ecological and evolutionary time scales (Louchart, 2008; Parmesan, 2006). Wide plasticity in migratory behavior is demonstrated by some individuals of a species initiating habitual seasonal migrations, with some foregoing migration to remain sedentary (Atwell et al., 2011). Understanding the mechanistic reasons for this difference in behavioral outcomes is important in predicting responses of migratory populations to a changing environment.

Efforts to model avian migration from an energetic perspective have necessarily been informed by empirical biological and physiological studies (see Malishev & Kramer‐Schadt, 2021 for a review). For many species these empirical studies elucidated relations between, for example: temperature and metabolism (Hartung, 1967; Klaassen, 1996; Smith & Prince, 1973), body mass and temperature (Baldwin & Kendeigh, 1938; Boos et al., 2007), and flight velocity and duration, and body fat content (Rayner, 1990). Using systems of equations to connect one facet to the next generates a series of expectations about the dynamics of migration (flight velocity, stopover frequency and duration, and survivorship) for a bird of a certain species and specific mass (Aagaard et al., 2018; Lonsdorf et al., 2016). Connecting approaches for predicting environmental effects on migration‐energetic dynamics with approaches evaluating the spatially explicit pattern of energetic‐based migratory movements can reveal how migration is affected by the distribution of forage material on the landscape. It can also inform how migration is likely to proceed given the differential expenditure of energy across the landscape and across temporally variable environmental conditions (Paxton et al., 2014).

While migration is a common term, we draw a distinction between it and movement and dispersal for consistency (Holloway & Miller, 2017). Movement is any change in location over time. Dispersal is movement that occurs in spatially limited areas without cyclical repetition (i.e., within a node—“nodes” here and throughout refers to an artificial grid of 20,053 cells with a resolution of 32.19 × 32.19 km overlaid on North America; Ai et al., 2012). Migration is predictable or routine seasonal movement among two or more distinct and consistent locations (Hansson & Åkesson, 2014). Migratory birds are faced with several contrasting strategies along their journey relating to timing, distance, velocity, altitude, and stopover length (Alerstam & Lindström, 1990). These dilemmas are captured by well‐studied tradeoffs: avoiding predation or refueling, flying at a speed allowing for maximum power or maximum range, and departing for migration early (to avoid inhospitable weather) or late (to further increase fat reserves) as opposed to “on time” (Alerstam & Lindström, 1990; Bruderer & Boldt, 2001; Drent et al., 2003; Hedenström, 1992; La Sorte et al., 2013; Pennycuick, 1975; Pennycuick et al., 2013; Pennycuick & Battley, 2003). Each trade‐off can be thought of as choice between different energetic or physiological strategies, essentially boiling down to prioritizing velocity or prioritizing energy‐efficiency. An easy analogy can be made between migrants and automobile drivers (Kitamura & Sperling, 1987); depending on the nature of the trip, a driver may choose to optimize for automobile velocity or fuel efficiency. Migratory birds must make similar tradeoffs during the course of their movements. For a more complete elaboration of the ecological processes of avian migration, see, for example, Aagaard et al. (2018), Alerstam and Lindström (1990), Alves et al. (2013), and Drent et al. (2003).

We extend existing energetics‐based models of waterfowl movement (Aagaard et al., 2018; Lonsdorf et al., 2016) to construct a full non‐breeding period model of waterfowl movement and energetics. Our model is of the type described by Malishev and Kramer‐Schadt (2021) and referred to as an energetics‐based Individual Based Model (eIBM); we note that the irreducible unit of interest in our models are more precisely considered “agents” rather than “individuals,” as we follow subsets of the population but not discrete individuals. The model approximates movements and stopovers as functions of weather, in terms of air temperature, air density, and snow depth, which, among other factors, influence the timing and extent of waterfowl migration (Nichols et al., 1983). The model begins in the late summer/early autumn as mallards (*Anas platyrhynchos*) are forced out of breeding habitat by inhospitable weather conditions. As in Aagaard et al. (2018) and Lonsdorf et al. (2016), we model bird movement as a function of the roosting quality (the proportion of landcover in an area classified as shoreline) and forage availability of each stopover site and the distance between the stopover site and departure site. We partition the population of mallards into a set of body condition classes based on body mass and body fat. We transition mallards among body condition classes based on differential movement and foraging, with the assumption of an inverse relationship between body condition and mortality risk.

We allow the bounds of the overwintering area to be an emergent property of the model rather than restrict it to static interpretations of historical overwintering grounds. We, therefore, add consideration of the distance from the stopover site to all other available stopover sites within an individual's flight range into our approximation of mallard movement. We also consider the consequences of a seasonally varying availability of forage by accommodating the consumption and natural decay of forage material. Additionally, we impose thresholds related to known waterfowl abundance–weather severity relations (Schummer et al., 2010) to mallard movement to restrict availability of the landscape to only those sites with hospitable conditions (Van Den Elsen, 2016). Weather severity can be thought of as a propellant during the early portion of the non‐breeding period to “push” mallards southward, while the breeding grounds serve as an attractant to “pull” mallards northward. In this way, mallards in this model tend to congregate along a weather‐severity isocline, staying as close to breeding grounds as weather conditions and metabolic demands allow (e.g., Robinson et al., 2016).

While the model structure outlined here is generalizable to all birds, we use dabbling ducks as an example (specifically, we parameterize our model for a mallard‐like, *Anas platyrhynchos*, dabbling duck). Mallards are exemplary model organisms in this context as they have been extensively studied in terms of their physiology and migratory dynamics (Krementz et al., 2011, 2012; Pennycuick et al., 2013; Prince, 1979) and are of great conservation and management interest (Heitmeyer, 2010).

An important advancement of this model is the development of over 60 years of migration trajectories using historical weather data to inform movement patterns. We also allow for sensitivity in input parameter values to approximate observed conditions. With this model, we seek to understand the influence of weather patterns and conditions, and *only* weather patterns and conditions, on the non‐breeding period of the annual cycle of migratory dabbling ducks. A full evaluation of this first‐principles exposition of avian migration requires broad‐scale data revealing avian migration patterns associated with historical environmental data. Our goal with this model was to evaluate our ability to predict a relationship between weather and migration patterns in the absence of such data. We can use our approach to determine if, all else being equal, variation present in a representative sample of observed historical environmental conditions facilitates demonstrable changes to avian migration patterns (Grimm & Railsback, 2012). Specifically, we expect the following as emergent properties of the model: (1) an explicit determination of the overwintering habitat; (2) a reduction in non‐breeding period mortality concurrent with a decrease in weather severity; and (3) to recover known migratory routes (flyways). Furthermore, we anticipate that our model can inform the following ecological hypotheses: (1) climate change has led to increased available habitat over time (and less severe weather) and (2) differences exist in avian response between mild and severe years comparable to historical weather reports.

## 2 METHODS

We discretized the landscape (North America) into 1036‐km^2^ stopover sites, or nodes. This spatial delineation is consistent with expectations that movement <16 km consists of dispersal within waterfowl (Beatty et al., 2014; Lonsdorf et al., 2016). Data availability and computational advancements allow us to greatly increase the temporal scale of the model relative to that considered in Lonsdorf et al. (2016). Rather than focus on five discrete migratory time slices we iterate across each day of the non‐breeding period. As such, our model begins after the molting stage of waterfowl (1 September of one calendar year) and terminates prior to the breeding period (31 May of the following calendar year). We use known values from the literature for physiological, anatomical, and metabolic dynamics to inform our model (Table 1).

**TABLE 1:** Definition, values, and units for each parameter used in the model

Parameter	Definition	Value	Units	Source
*D*	Local movement distance	16,000	m	Beatty et al. (2014)
*M*	Body mass	800–1300	grams	Owen and Cook (1977)
*Ws*	Wing span	0.95	m	Drilling et al. (2020)
*Wa*	Wing area	0.1	m^2^	Bruderer and Boldt (2001)
*E*	Energetic cost of flight (per hr)	0.042–0.076	kJ/h	Pennycuick (2008)
*N*	Node size resolution	1036	km^2^	Lonsdorf et al. (2016)
*N_0_*	Initial population size	19,856,514	individuals	—
*s_d_*	Daily survivorship range	0.9975–0.9997	—	Lonsdorf et al. (2016)
*f*	Proportional body fat	0–0.14	—	Dabbert et al. (1997), Boos et al. (2007)

### 2.1 Model operation

The workflow to simulate daily movement was constructed to closely approximate the actual processes of movement, dispersal, and migration while operating under the constraints of a sequential modeling framework. Our pattern proceeded iteratively as:

Forage →Departure → Arrival →Mortality → Forage…

**FIGURE 1:**
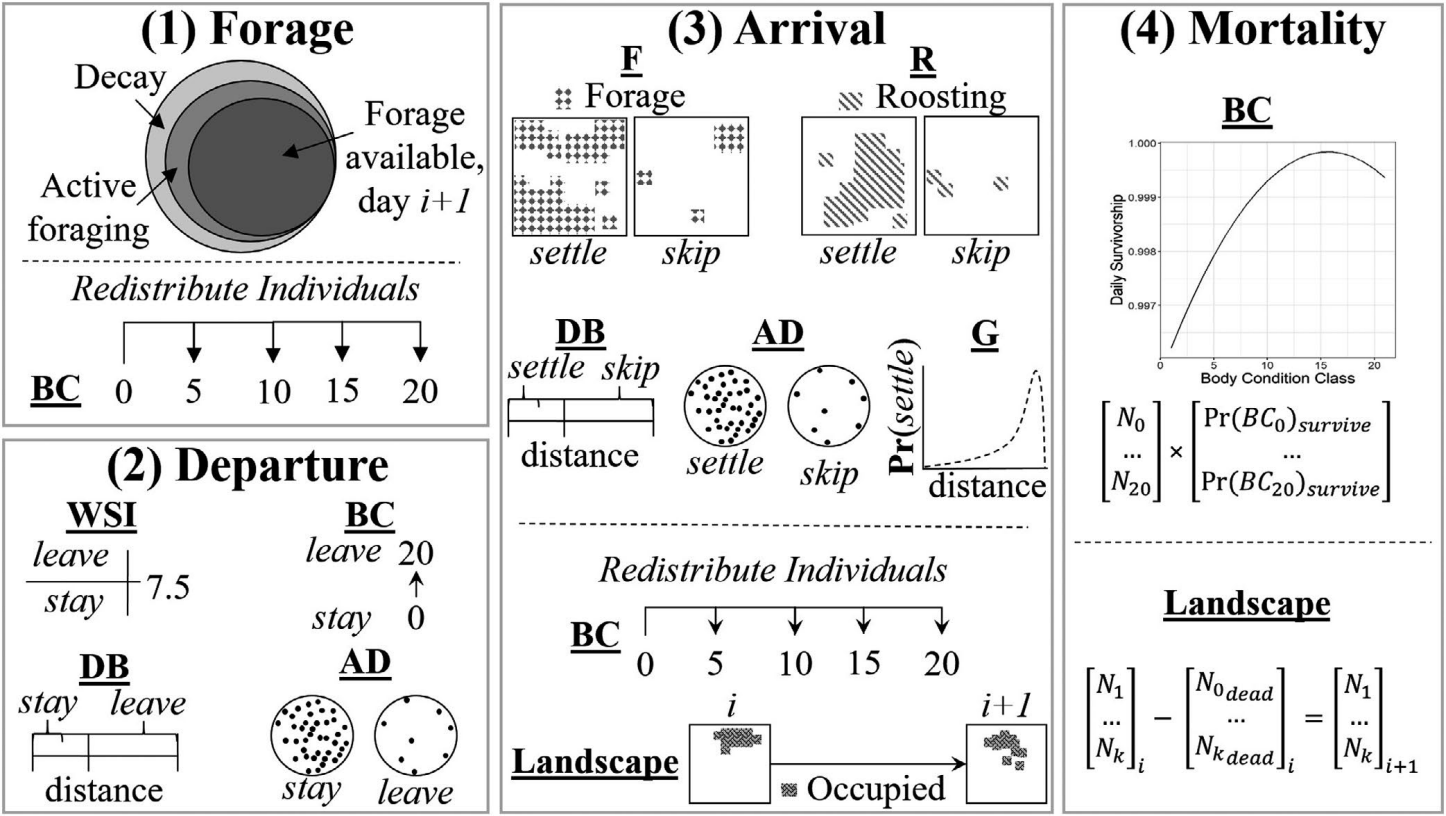
Graphical representation of the order of operations of the model. We initiated (1) foraging activity (i.e., the loss of forage material from the habitat as a result of active foraging and natural decay and the subsequent acquisition of the actively foraged material to augment body condition, BC) prior to (2) departure, the probability of which was dictated by the node‐specific weather severity index (WSI), class‐specific BC, distance between a focal node and the nearest breeding node (distance to breeding grounds, DB), and the node‐specific air density (AD). Arrival of individuals (3) followed, informed by node‐specific forage quantity (*F*), roosting habitat quality (*R*), cumulative gamma‐movement probability (the probability of moving between each pair of nodes on the landscape, given the distance between them; G), as well as AD and DB. Individuals were then redistributed among BC classes according to energy expended in flight and were redistributed spatially based on to‐from node flights. Finally, the population incurred mortality (4) according to survivorship rates related to each BC. We calculated the number of individuals per body condition after mortality and arrival, as well as the number of individuals per node, for the following day

Within each component of our model there were secondary procedures invoked, for example, to effectively allocate forage material among individuals within the population and to distribute individuals across the landscape according to the spatial pattern of high‐quality habitat. The overall workflow is depicted in Figure 1. R code (R version 3.9.5, R Core Team, 2018) is available in File S1.

#### 2.1.1 Forage

A simulated day began with foraging, a weather‐ and body condition‐dependent process, which transfers energy from forage material on the landscape into energy as fat to individual mallards. Individuals in each node (distributed spatially according to NatureServe range maps [Ridgley et al., 2005] and breeding population survey data; see Lonsdorf et al., 2016) were allocated among 21 body condition classes, with higher classes representing better body conditions. Body condition is defined by body fat (the product of body mass and a sequence of body fat proportions from 0% [body condition 0, fatal] to 11%; as stated subsequently, we varied the upper limit of body fat among 8%, 11%, and 14% to assess the sensitivity of migration to this upper limit). An individual's ability to accumulate forage—its rate of daily gain (DG)—was a function of disturbance (degree of urbanization in a node, as a proxy to human activity), fuel deposition rate (FDR, kJ per day), and temperature‐dependent basal metabolic rate (Aagaard et al., 2018). We summed the fuel deposition of each individual to calculate the amount of energy removed from the landscape in each node as a result of active foraging.

To calculate the rate of natural decay (*D*) in forage material we multiplied the amount of forage material (*E*, kJ) present in each vegetative community (shoreline, crop, wooded wetland, herbaceous wetland, Table 2; Center for Topographic Information, Earth Sciences Sector and Natural Resources Canada, 2009; Fry et al., 2011—see Appendix S2 for details on land cover data) by a land cover‐specific decay rate (*r*) (derived from Fredrickson & Reid, 1988; Heitmeyer, 2010) to the power of the day of the non‐breeding period (*i*):

$$D=E\times r^{i}.$$

**TABLE 2:** Values assigned to the weights for natural forage decay rates in available land cover classes

Land cover class	Weight
Shoreline	0.9998
Crops	0.9970
Woody wetlands	0.9965
Herbaceous wetlands	0.9910

We calculated the total forage available on the landscape in the next time step by subtracting the amount of forage material subject to decay and active foraging from the total amount of forage available (*F*) at the outset of day *i*,

$$F_{i+1}=F_{i}‐D‐\sum \text{FDR}.$$

#### 2.1.2 Departure

Each day, individuals must decide whether to stay and continue foraging or depart from a node. We assumed that individuals in poorer body condition experiencing mild weather nearest to breeding nodes with potential for high rates of gain of body mass were less likely to leave a node, instead remaining to continue foraging, while an individual in better body condition facing severe weather far from breeding nodes with potential for low rates of gain of body mass were highly likely to leave. The probability of individuals remaining in a given node or departing from it depends on each individual's body condition (BC), the weather severity index (WSI) it faces within the node, the distance between the origin node, its current location, and the nearest breeding node (DB), and the disturbance‐dependent daily gain in body mass (DG). The components were combined according to the follow equation:

$$\sum \mathrm{Pr}\left(\text{depart}\right)=\mathrm{Pr}\text{(depart}\text{|WSI})+\mathrm{Pr}\text{(depart}\text{|BC})+\mathrm{Pr}\text{(depart}\text{|DB})+\mathrm{Pr}\text{(depart}\text{|DG}).$$

Three of the four components, BC, WSI, and DB, were each calculated using a Monod function (Tjørve, 2003), which produced a saturating curve for the target effect, with exponents applied to vary the shape of the resulting curve. Daily Gain was calculated using a linear decreasing function. The discrete probabilities of departure had the following forms:

$$\mathrm{Pr}\left(\text{depart|WSI}\right)=\left(\frac{{\left(\text{WSI}+7.5\right)}^{3}}{{\left(\text{WSI}+7.5\right)}^{3}+{\left(7.5\right)}^{3}}\right),$$

$$\mathrm{Pr}\left(\text{depart|BC}\right)=\left(\frac{{\text{BC}}^{8}}{{\text{BC}}^{8}+{\left(N_{\text{BC classes}}‐3\right)}^{8}}\right),$$

$$\mathrm{Pr}\left(\text{depart|DB}\right)=\left(\frac{{\text{DB}}^{5}}{{\text{DB}}^{5}+{\text{flight range}}^{5}}\right),$$

$$\mathrm{Pr}\text{(depart}\text{|DG})=\left(\frac{1‐\left(\text{FDR +}\;1\right)}{5}\right).$$

The values for the exponents in each of these three cases were selected to generate reasonable curves for each probability of departure component. We assessed what constitutes “reasonable” based on the reaction in the model to changes in these values. We expected that the curve for the body condition component would have a pronounced inflection point to represent a high probability of departure for mallards in the highest body conditions (top three), and a relatively low probability of departure for mallards in moderate to low body conditions. Weather severity, in contrast, was expected to be relatively more linear. Thus, we set the exponents in body condition to be nearly three times as great as those in WSI.

More formally, we calculated the number of individuals, *N*, departing each node (*N[*depart*]*) as the product of the abundance per node, *i*, on day *k* (*d_k_*), and the proportion of individuals in each BC, *j*; this product was then multiplied by the BC‐dependent probability of departure:

$$\left[\left[\left[\begin{array}{c} d_{k}\left(N_{1}\right)\\ \ldots \\ d_{k}\left(N_{i}\right) \end{array}\right]\times \left[\begin{array}{ccc} {\text{BC}}_{1} & \ldots & {\text{BC}}_{j} \end{array}\right]\right]\times \left[\begin{array}{ccc} \text{Pr}{\left(\text{depart}\right)}_{1} & \ldots & \text{Pr}{\left(\text{depart}\right)}_{j} \end{array}\right]\right]=\left[\begin{array}{c} d_{k}\left(N{\left[\text{depart}\right]}_{1}\right)\\ \ldots \\ d_{k}\left(N{\left[\text{depart}\right]}_{i}\right) \end{array}\right]$$

#### 2.1.3 Arrival

Once individuals choose to depart, they must decide how far to fly and where to land. How far mallards can fly is a function of body condition (mass, body fat proportion), flight cost, and flight velocity. Using the relations set forth in the program Flight (for Windows, version 1.25 [https://booksite.elsevier.com/9780123742995/casestudies/04~Flight_1.25_ReadMe.txt]; Pennycuick, 2008), we calculated the chemical power, velocity for maximum range, and effective lift‐to‐drag ratio for mallards from a distribution of available body masses (Owen & Cook, 1977), wing spans (Drilling et al., 2020), wing areas (Bruderer & Boldt, 2001), and at various air densities, across a range of potential true air speeds. These calculations led to the ultimate output of the range of flight costs (kg of fat metabolized per km) and flight velocities (km per h), for the input range of morphometric features. Flight velocity ranged from 80 to 98 km/h, and flight cost ranged from $1.98\times 10^{‐5}\;\text{to}\;1.71\times 10^{‐4}$ kg/km. The code used to generate these calculations is included in File S1.

Mallards select for stopover sites according to WSI (Schummer et al., 2010), among other factors (e.g., forage availability, roosting habitat, and distance to breeding grounds; see Beatty et al., 2017). We assume, on average, mallards will tend to select more attractive nodes, that is, nodes with low WSI and higher air density (AD), plentiful forage availability and roosting habitat (*R*), nearer to breeding grounds, and within the flight range (defined as a node‐specific gamma movement probability, *G*; the cumulative probability of moving from a node to all other nodes in the landscape based on the distance between each pair of nodes). We used a Cobb–Douglas function to define node attractiveness as an optimum of the input factors (Brown & Robinson, 2006).

**FIGURE 2:**
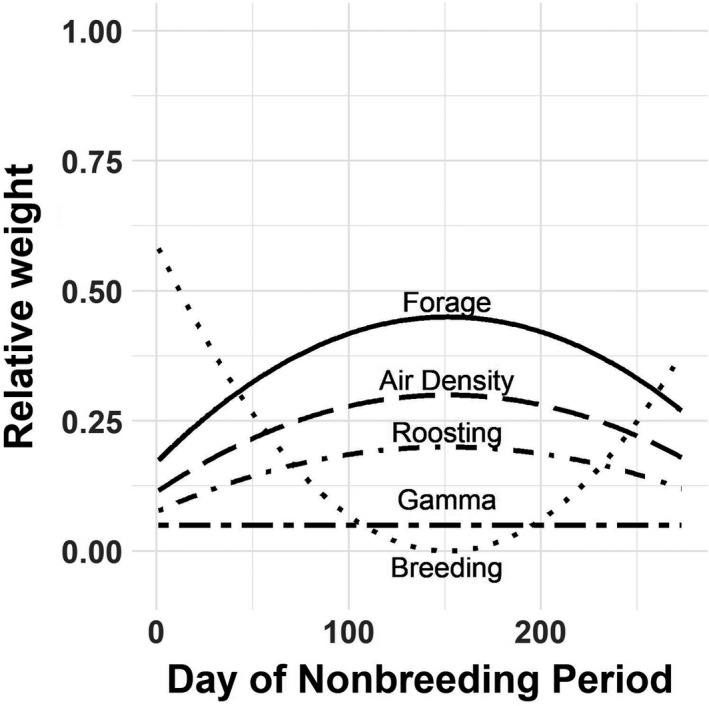
Graphical representation of exponential weights for each of the four components of the Cobb–Douglas function used to define node attractiveness over time: amount of forage availability, air density, proportion of roosting habitat in each node, distance to the nearest breeding node, and a cumulative probability of moving to a node from all other nodes based on a gamma function

We restricted movement to nodes in which the WSI was below the empirically derived threshold (7.5; Schummer et al., 2010). The remaining five components of the arrival function (forage, roosting, air density, breeding ground distance, and gamma movement probability) were individually weighted to allow for the differential significance of particular parameters ($w_{f}$, forage availability; $w_{r}$, roosting quality; $w_{a}$, air density; $w_{b}$ distance to nearest breeding node; and $w_{g}$, node‐specific gamma movement probability). We also assumed variable relative importance of each component over time (Figure 2). We assumed that distance to the nearest breeding node was the most important consideration for migrants proximal to the breeding period (i.e., early and late in the non‐breeding period). We assumed that forage availability and roosting quality increased in importance up to the mid‐winter period of the non‐breeding period—with forage increasing more so than roosting—and then decreased to initial values again by the end of the non‐breeding period. We held the node‐specific gamma movement marginal probability steady across the non‐breeding period because the probability of moving between any given pair of nodes (depending only on the distance between them) is not expected to vary temporally.

Input values for the component weights were, for $w_{f}$, 0.45; $w_{a}$, 0.3; $w_{r}$, 0.2; and $w_{g}$, 0.05. The weight for the distance to breeding grounds, $w_{b}$, was set to the sum of the daily weights for all other components subtracted from one. To establish the structure of the weights for the components assumed to have nonlinear dynamics, we randomly selected a date during the mid‐winter phase of the non‐breeding period (between 31 December and 31 January) to serve as our inflection point. We then generated a sequence of values, *c*, from 1 to 0.1 to 1, with an inflection occurring on the specified day of the non‐breeding period, *d*, for use in a function to calculate the corresponding weights for each component:

$$w_{f}=‐\left(0.9\times \left({\left(c_{d}‐\left(\frac{c_{d}}{n}\right)\right)}^{2}\right)\right)+0.45$$

$$w_{a}=‐\left(0.6\times \left({\left(c_{d}‐\left(\frac{c_{d}}{n}\right)\right)}^{2}\right)\right)+0.3$$

$$w_{r}=‐\left(0.4\times \left({\left(c_{d}‐\left(\frac{c_{d}}{n}\right)\right)}^{2}\right)\right)+0.2$$

$$w_{g}=0.05$$

$$w_{b}=1‐\sum \left(w_{f},w_{a},w_{r},w_{g}\right),$$

where n is the length of the non‐breeding period in days.

Using this process to generate the daily weights, the full Cobb–Douglas function for the probability of arrival in a given node was defined as:

$$A=F^{w_{f}}\times {\text{AD}}^{w_{a}}\times R^{w_{r}}\times {\text{DB}}^{w_{b}}\times G^{w_{g}}.$$

Each of these components was normalized to a 0–1 scale, using $x‐\text{min}\left(x\right)/\min\left(x\right)‐\text{max}\left(x\right)$; the node with the greatest amount of forage availability on a given day was assigned a normalized forage availability score of 1; we repeated this calculation for roosting quality, distance to nearest breeding node, and node‐specific gamma movement probability.

#### 2.1.4 Mortality

We assumed that individuals in poorer body conditions had higher daily rates of mortality than individuals in better body conditions (Bergan & Smith, 1993; Davis et al., 2011)—in keeping with the assumption of increased mortality with increased energy deficits (e.g., Lonsdorf et al., 2016). Each day we multiplied the survivorship associated with a given body condition by the number of individuals in that body condition class. For the purposes of this study, hunting‐harvest is not considered as a mortality source because the model is only testing weather effects.

### 2.2 Daily abundance

We redistributed individuals across the landscape and among body condition classes according to their probabilities to stay/depart and arrive. We calculated the following day's abundance in a node as the product of the total number of individuals departing all nodes and the probability of arrival in the node:

$$\left[\begin{array}{c} D_{k}\left(N{\left[\text{depart}\right]}_{1}\right)\\ \ldots \\ D_{k}\left(N{\left[\text{depart}\right]}_{i}\right) \end{array}\right]\times \left[\begin{array}{c} Pr{\left(\text{arrive}\right)}_{1}\\ \ldots \\ Pr{\left(\text{arrive}\right)}_{i} \end{array}\right]=\left[\begin{array}{c} D_{k}\left(N{\left[\text{arrive}\right]}_{1}\right)\\ \ldots \\ D_{k}\left(N{\left[\text{arrive}\right]}_{i}\right) \end{array}\right],$$

added to the difference of the current abundance and the number of individuals departing the node.

We computed the number of individuals departing a node in each body condition and the number of individuals remaining in a node in each body condition. We decremented the body condition of departing individuals according to the distance between origin‐node and destination‐node, using established relations for the mass‐dependent cost of flight per unit distance (e.g., see Aagaard et al., 2018). This decrement‐function informed the number of individuals arriving in each node in each body condition, which we used to calculate the number of individuals in each node in each body condition class on the following day.

The final abundance for a given node on the following day was the abundance in that node on the current day minus the number of individuals that died in that node on that day. Taken together with *Arrival*, this produced:

$$\left[\left(\left(\left[\begin{array}{c} D_{k}\left(N_{1}\right)\\ \ldots \\ D_{k}\left(N_{i}\right) \end{array}\right]‐\left[\begin{array}{c} D_{k}\left(N{\left[\text{depart}\right]}_{1}\right)\\ \ldots \\ D_{k}\left(N{\left[\text{depart}\right]}_{i}\right) \end{array}\right]\right)+\left[\begin{array}{c} D_{k}\left(N{\left[\text{arrive}\right]}_{1}\right)\\ \ldots \\ D_{k}\left(N{\left[\text{arrive}\right]}_{i}\right) \end{array}\right]\right)\times \left[\begin{array}{ccc} {\text{BC}}_{1} & \ldots & {\text{BC}}_{j} \end{array}\right]\right]‐\left[\begin{array}{ccc} Pr{\left(\text{die}\right)}_{1} & \ldots & Pr{\left(\text{die}\right)}_{j} \end{array}\right]=\left[\begin{array}{c} D_{k+1}\left(N_{1}\right)\\ \ldots \\ D_{k+1}\left(N_{i}\right) \end{array}\right].$$

### 2.3 Parameterization

For some parameters in our model there is a lack of empirical evidence to inform their value. We defined these parameters probabilistically, as a function of body condition, to allow for sensitivity in the model (Table 1; see also Appendix S1 for definitions of parameters and distributions). These parameters included daily survivorship, flight power components, and energetic costs of flight. We also allowed parameters with known individual variation to vary within the population, such as flight velocity, body mass, and proportion of body mass composed of metabolizable lipids. We applied these distributions to the starting population of ~20 million mallards and updated the fluctuating variables according to incurred energetic costs (body mass, available metabolizable lipids). This arrangement allowed us to capture a realistic representation of the distribution of realized values for each parameter in the model without unreasonably increasing computation time. We varied the parameter values associated with the prior distributions for morphological features to evaluate their effects on the model. We monitored estimated survivorship as a comparator, as well as daily survivorship, flight power components, morphological components, and energetic components.

#### 2.3.1 Daily survivorship

Daily survivorship ranged from 0.99620 to 0.99984, from the second body condition bin to the optimal body condition bin (body condition bin 1 represented dead individuals, survivorship =0). We assumed mallards that exceeded some critical mass would experience heightened mortality as a result of increase predation risk (or decreased predation avoidance ability), in keeping with optimal body mass theory (Lima, 1986). We therefore set the “optimal” body mass to be that of a 1.625 kg mallard (~0.134 kg of fat), about the maximum of observed mallard body masses in the field (Owen & Cook, 1977; while noting and allowing for the rare occurrence of heavier mallards).

#### 2.3.2 Flight power components

Air density was informed by measured and interpolated air pressure values, as detailed in Appendix S1. The absolute range in air density across the period of sampled data was 0.95–1.30 kg/m^3^. The true air speed—that is, the velocity at which molecules *appear* to move past a moving body from the perspective of the body in motion—ranged from 10 to 25 m/s. Flight velocity (the actual velocity of the body in motion) ranged from 75.92 to 87.37 m/s, according to relations laid out in Flight (for Windows, version 1.25 [https://booksite.elsevier.com/9780123742995/casestudies/04~Flight_1.25_ReadMe.txt]; Pennycuick, 2008).

#### 2.3.3 Morphological components

Body mass, wing span, and wing area were all modeled to follow skew‐normal distributions with *μ* = 1.2 and *σ* = 1.21 [body mass, *kg*, Owen & Cook, 1977; assuming most individuals begin migration only when closer to optimal body condition], *μ* = 0.95 and *σ* = 1 [wing span, *m*, Drilling et al., 2020], and *μ* = 0.1 and *σ* = 0.1 [wing area, *m^2^*, Bruderer & Boldt, 2001]. These values were used in the program Flight (Pennycuick, 2008), as described above.

The proportion of body mass composed of metabolizable lipids (*kg*) was set to 11%, and was subsequently allowed to vary from 8 to 14% (using values from Boos et al., 2007; Dabbert et al., 1997). Lipids account for ~81% to 84% of metabolizable energy (Boos et al., 2007). Not all lipids are available for metabolic processes (some are retained for other purposes, not detailed; Boos et al., 2002, 2007). We assume that the ~16% to 19% of metabolizable energy provided by sources other than lipids is used for processes other than flight (basal metabolic rate, reproductive organs, cellular replacement, etc.). Therefore, we assume that all energy directed toward powered flight relies on lipids as its source exclusively, and not all the ~10% to 16% of body mass composed of lipids is available for powered flight processes.

Body mass had a mean of 1.2 kg (Owen & Cook, 1977; Pennycuick, 2008) and a distribution with a range of 0.5–2 kg. Wing span and wing area had ranges of 0.75–1.15 m and 0.09–0.11 m^2^, respectively (Bruderer & Boldt, 2001).

#### 2.3.4 Energetic components

The cost (kg of body fat) per unit distance flown (km) ranged from $2.1\times 10^{‐5}$ to $1.6\times 10^{‐4}$, according to relations laid out in Flight (Pennycuick, 2008). Without clear guidance from the literature to inform a consistent relationship between fuel deposition rate and climatic factors, we defined the coefficient of fuel deposition rate as a multiple of body mass and set it to 1.1% initially, and subsequently set it to 0.5 and 2 to represent low and high values. This arrangement is in line with values presented by Lindström (2003), in which the maximum fuel deposition rate for a ~1‐kg non‐passerine bird caps out at 2% of the lean mass, with a minimum of 0.3%.

### 2.4 Migration paths

**FIGURE 3:**
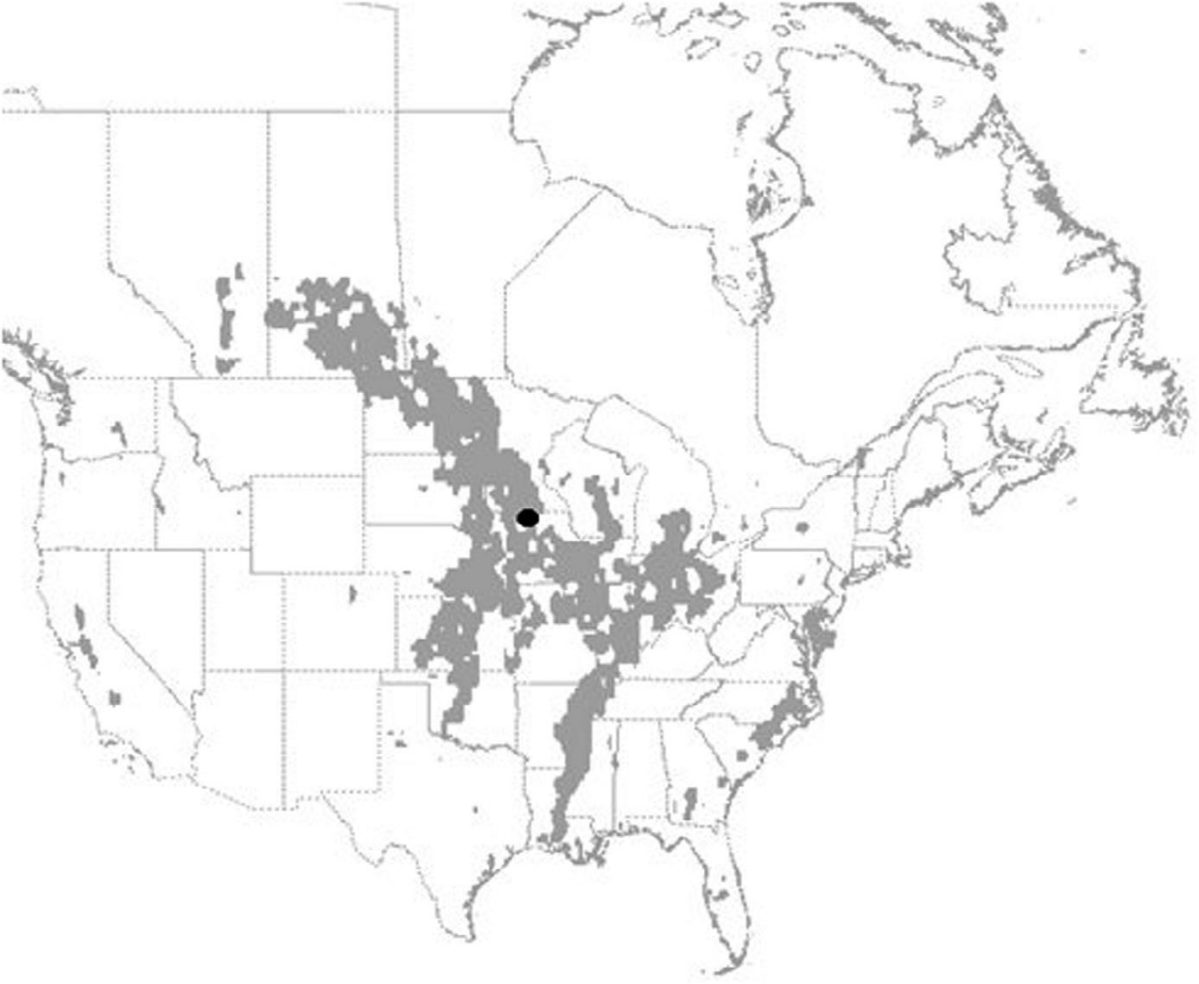
An example of the abundance‐weighted center‐of‐mass for the population on the 50th day of migration, represented by the black dot. The gray shaded areas represent the top 2% most populous nodes on day 50

For each year of the simulation we recorded a migration “path”—an approximation of the median route taken by the population during the non‐breeding period. We computed the abundance‐weighted center‐of‐mass for the population on each day; given the spatial distribution of individuals within nodes across the landscape, we identified the point representing the centroid of the population (Figure 3). By tracking the latitude of this point each day we assembled a trajectory representing the latitudinal and longitudinal shift of the centroid of the population throughout the non‐breeding period. Evaluating the nadir of the latitudinal shift across years informs potential temporal patterns in migration and overwintering dynamics. For example, one might expect a general northward regression of the southern‐most point of the population center‐of‐mass through time as average global atmospheric temperatures increase (Aagaard et al., 2018). Alternatively, the southern‐most point might be more closely related to weather severity, with a changing climate leading to increasingly variable weather patterns from one year to the next; as such, there may not be a consistent decrease in severe weather but rather more frequent extremes (more unusually mild and unusually severe weather years). By regressing the southern‐most point of the population center‐of‐mass with year and WSI we can potentially parse this difference.

We calculated the mean distance between all consecutive population centers‐of‐mass, as well as the distance between the northern‐most and southern‐most population centers‐of‐mass. These metrics informed the mean distance moved by the population from one day to the next, as well as the separation between breeding grounds and overwintering habitat. We identified the most commonly used nodes during the course of the non‐breeding period by measuring the total abundance in each node on each day to compute the top 2% most populous nodes per day. We used these metrics to produce animations for each year of the record showing the daily, normalized (0–1) abundance for each node in the landscape.

### 2.5 Data sources

Our model takes as input six data layers relating to habitat state and weather conditions. There are five layers relating to habitat state; the first was derived from National Land Cover Database (NLCD) 2006 for the USA (Fry et al., 2011) and the CSC2000v for Canada (Appendix S2) to estimate roosting and foraging quality. We used NatureServe range maps (Ridgley et al., 2005) to identify potential starting locations among which to distribute mallards at the onset of the non‐breeding period. We used these input layers in conjunction with breeding population survey data (U.S. Fish and Wildlife Service, 2013a, 2013b) to establish abundance at breeding sites by weighting the total number of mallards by the quality of habitat within the site and the breeding population survey results for the area. We relied on daily climate data from the National Oceanic and Atmospheric Association's National Centers for Environmental Prediction National Center for Atmospheric Research Reanalysis Project (NOAA NCEP; Kalnay et al., 1996).

We considered sites with a higher proportional area of shoreline, herbaceous wetlands, and wooded wetlands to be of higher quality for roosting. We stress that shoreline here is used as a proxy of availability. Because wetlands are heterogeneously and unevenly mapped across the continent, we used shoreline from river line files and waterbodies to serve as an approximation of roost availability. In this fashion, breeding sites of high quality aligning with large numbers of mallards from the breeding population survey hosted the greatest numbers of individuals. We estimated mean forage availability per site (in 0.1 GJ units) at the outset of the non‐breeding period based on an evaluation of land cover using a range of parameter estimates informed by literature (Fredrickson & Reid, 1988; Heitmeyer, 2010; Lonsdorf et al., 2016). Further details of this process are available in Lonsdorf et al. (2016) and summarized in the File S3; the values used for the forage availability parameter estimates are provided in Lonsdorf et al. (2016, Appendix C).

### 2.6 Landscape generation

Mallards have documented preferences for wetlands with shallow water (5–20 cm) in which to forage and near which to roost (Colwell & Taft, 2000; Guillemain et al., 2000). We classified shoreline cover as available roosting habitat (i.e., with a value of 1, while all other cover types are 0), and calculated the proportion of each node occupied by shoreline cover. Multiplying this proportion by the area of the focal node yielded the value of roosting availability provided by that node.

We multiplied the amount of forage provided by each land cover type represented within the node (using food‐habit information from the literature; see Lonsdorf et al., 2016) by the proportion of the node classified as each land cover type. We multiplied this value by the proportion of forage *available* in a node, based on the distance to the nearest roosting site. Areas in which forage and roosting habitat were nearby had greater proportions of their forage available for consumption to account for a decrease in net energy extracted from a node given the increased distance traveled to the foraging sites within the node. We multiplied this roosting distance‐ and area‐dependent forage availability measure by the area of the node to calculate the quantity of forage available in each node (Beatty et al., 2014; Pearse et al., 2012). See File S2 for more details on this process.

Crucially, and only for the purposes of this study, land‐use change is not considered here because the model is only testing weather effects. Therefore, the above methods and details provided in the Supporting Information are used to create a *static* landscape depiction of forage and roosting availability.

### 2.7 Winter severity

To quantify the severity of the weather in a given node (and thus the probability that mallards will occupy that node), we followed the framework of Schummer et al. (2010) wherein a weather severity index (WSI) was calculated based on the depth of snow in a node (*S*, cm), the number of consecutive days with snow depth ≥2.54 cm (*S*_days_), the temperature in a node (*T*, °C), and the number of consecutive days with temperature <0 (*T*_freeze_). The formulation follows:

$\text{WSI}=\left(S\times 0.394\right)+S_{\text{days}}+\left(‐T\right)+T_{\text{freeze}}.$

Schummer et al. (2010) found that the rate of change of the relative abundance at a location switched from positive to negative when WSI = 7.5; we invoked this value as the threshold below which individuals were expected to remain in a node and above which individuals were expected to depart. The code used to generate these calculations is included in File S3.

Landscapes change in space over the course of seasons and years; rainfall, for instance, can flood rivers and agricultural fields, altering patterns in roost and forage availability. For our purposes here, we allow a much narrower set of seasonal changes to the landscape. Snow and ice cover impede access to calories. In areas where calories are accessible, the caloric landscape is allowed to change over the course of the season as calories degrade or are consumed. We presume no other changes to the landscape to control for potential land cover effects when evaluating consequences of weather conditions.

### 2.8 Model evaluation

We monitored a suite of metrics as we iterated our simulation across years to evaluate the degree to which migratory patterns differed annually. We monitored non‐breeding period mortality (*N_0_ – N_n_*; *n* is the last day of the non‐breeding period). We also monitored landscape availability, based on the number of nodes in which the WSI was less than the threshold each day. We calculated the mean availability (and standard deviation) of hospitable area, as well as the minimum availability at any point during the non‐breeding period. These values informed the average weather severity across the non‐breeding period, and the severity of the weather during the least hospitable portion of the non‐breeding period. We ran the model in R (R Core Team, 2018) relying most heavily on data.table (Dowle & Srinavasan, 2021) and the suite of packages from tidyverse (Wickham et al., 2019).

## 3 RESULTS

### 3.1 Objectives

**FIGURE 4:**
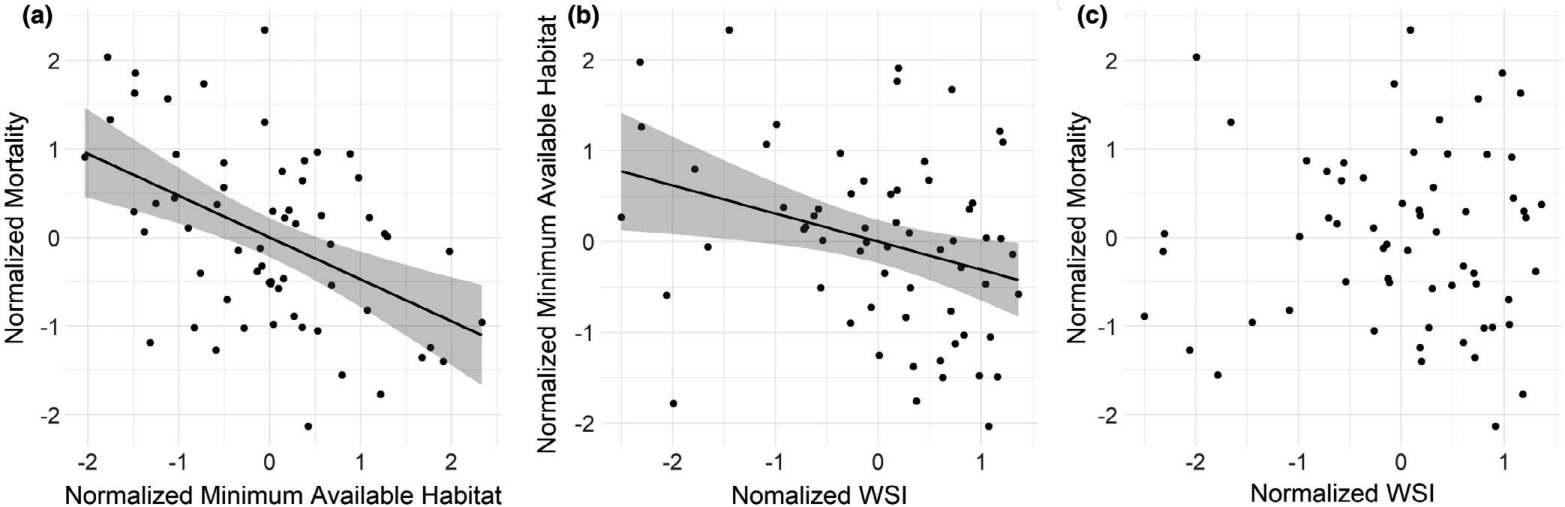
(a) Normalized $\left(x_{i}‐\bar{x}/\text{SD}\left(x\right)\right)$ minimum available habitat on the landscape as a function of normalized weather severity index (WSI); (b) Normalized mortality during the non‐breeding period (September to May) as a function of normalized minimum available habitat; and (c) Normalized mortality as a function of normalized WSI. Black lines indicate the line of best fit of a generalized linear model and associated standard error (gray shaded area)

**FIGURE 5:**
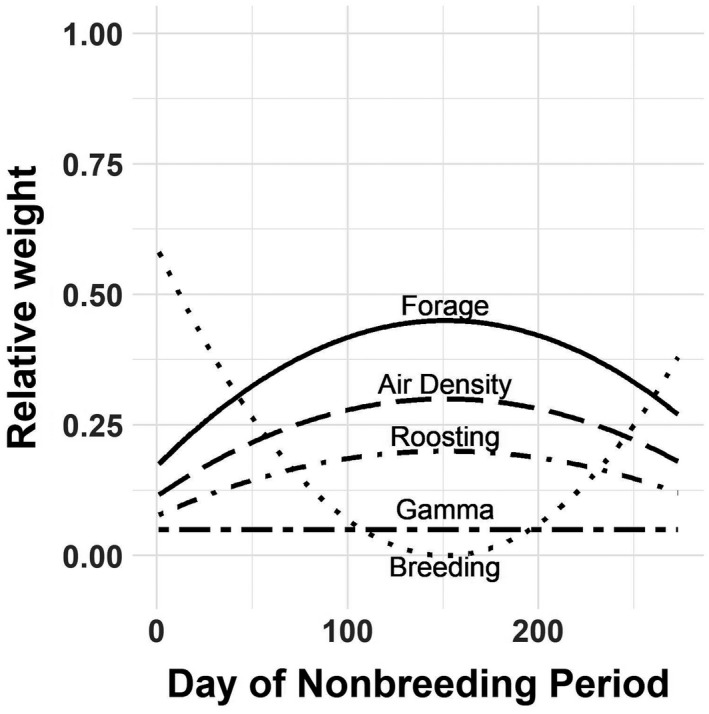
(a) Mean weather severity index (WSI; Schummer et al., 2010) within the available habitat (areas with WSI < 7.5) and (b) across the entire landscape showed differing patterns over time

**FIGURE 6:**
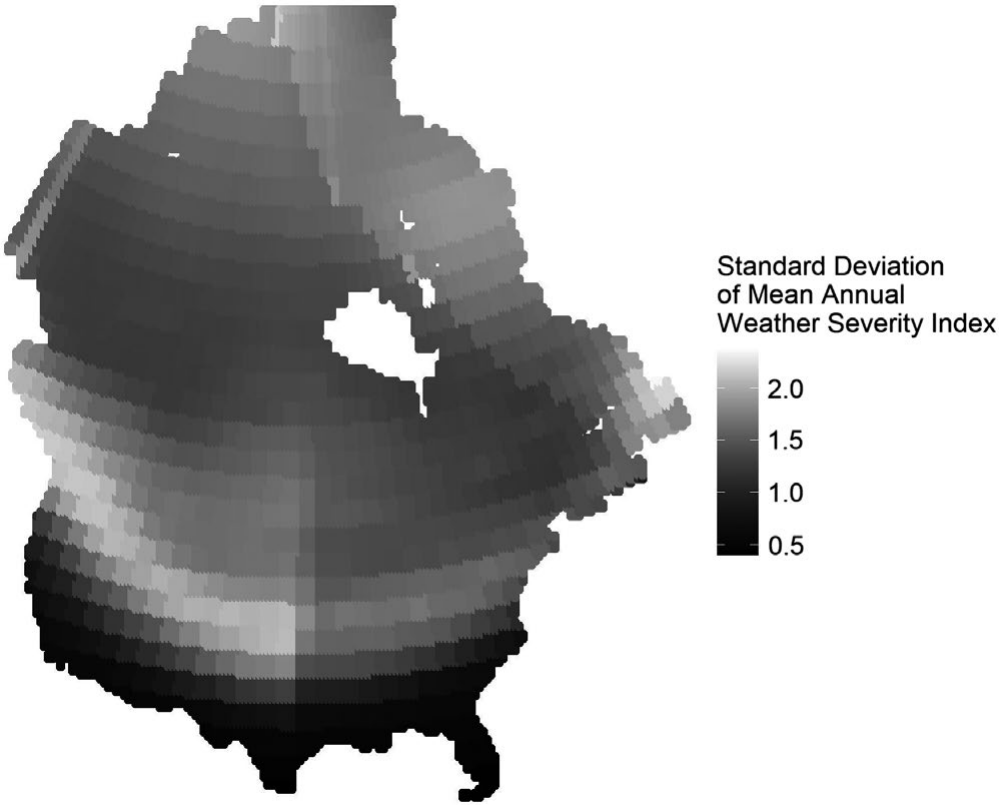
The standard deviation in the annual mean weather severity index (WSI) over the period of record (1957–2019) for each node in North America. Mid‐latitude and above areas were subject to greater variation in weather severity over time than southerly areas, which are more consistently incorporated in “available habitat” (areas with WSI < 7.5)

As expected, we observed weather patterns affecting the non‐breeding period of the annual cycle of migratory mallards. We were able to discern overwintering habitat as an emergent property of the model (Figure 3). The average proportion of available habitat (WSI < 7.5) across the landscape increased as winter weather severity decreased (Figure 4a), and mortality decreased as the proportion of available habitat increased (Figure 4b). Unexpectedly, mortality did not demonstrate any correlative trend with weather severity (Figure 4c), perhaps because mallards flew beyond the range of the affected area. Importantly, whereas we summarized weather severity across the entire landscape, there was spatial heterogeneity in the variation of weather severity. The summarized WSI in the available habitat showed a slight increase over time, with a few years of above‐average weather severity later in the record (especially 2009–2010 and 2010–2011). However, the summarized WSI across the entire landscape decreased more dramatically over the same timeframe (even 2009–2010 and 2010–2011 produced below‐average WSI scores) (Figure 5). We plotted the standard deviation of the mean annual WSI for each node across the period of record to demonstrate this point (Figure 6).

**FIGURE 7:**
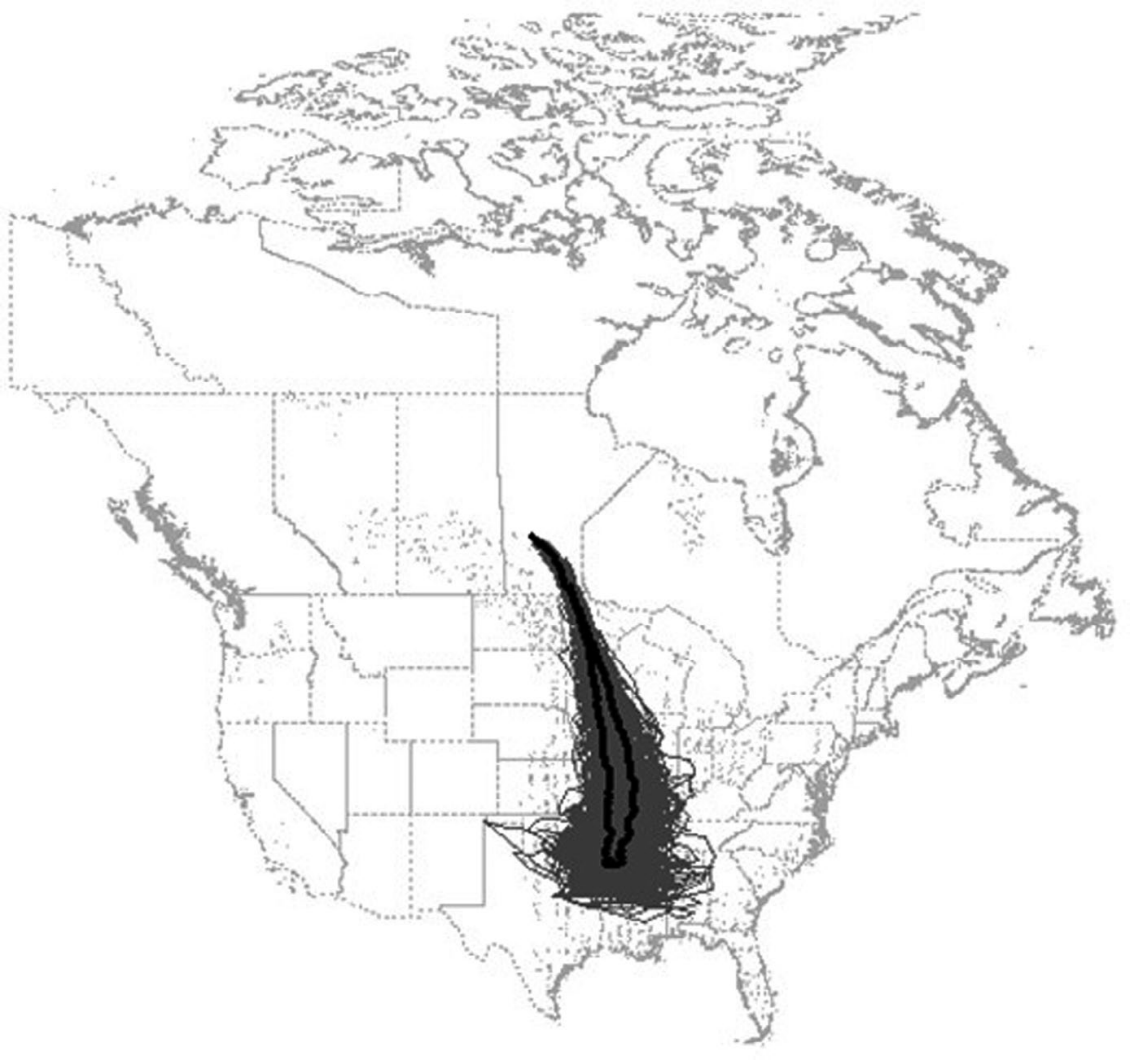
Map showing the 2% most populous nodes, in gray, across the non‐breeding period (September to May) for all years (1957–2019). The darker the gray the more often a node occurred within the 2% most populous nodes across the record. The 2% most populous nodes were similar across most years, hence the consistent patches. Lines represent the path of the abundance‐weighted population center of mass, or migration route, across years, with the mean of all years in black

**FIGURE 8:**
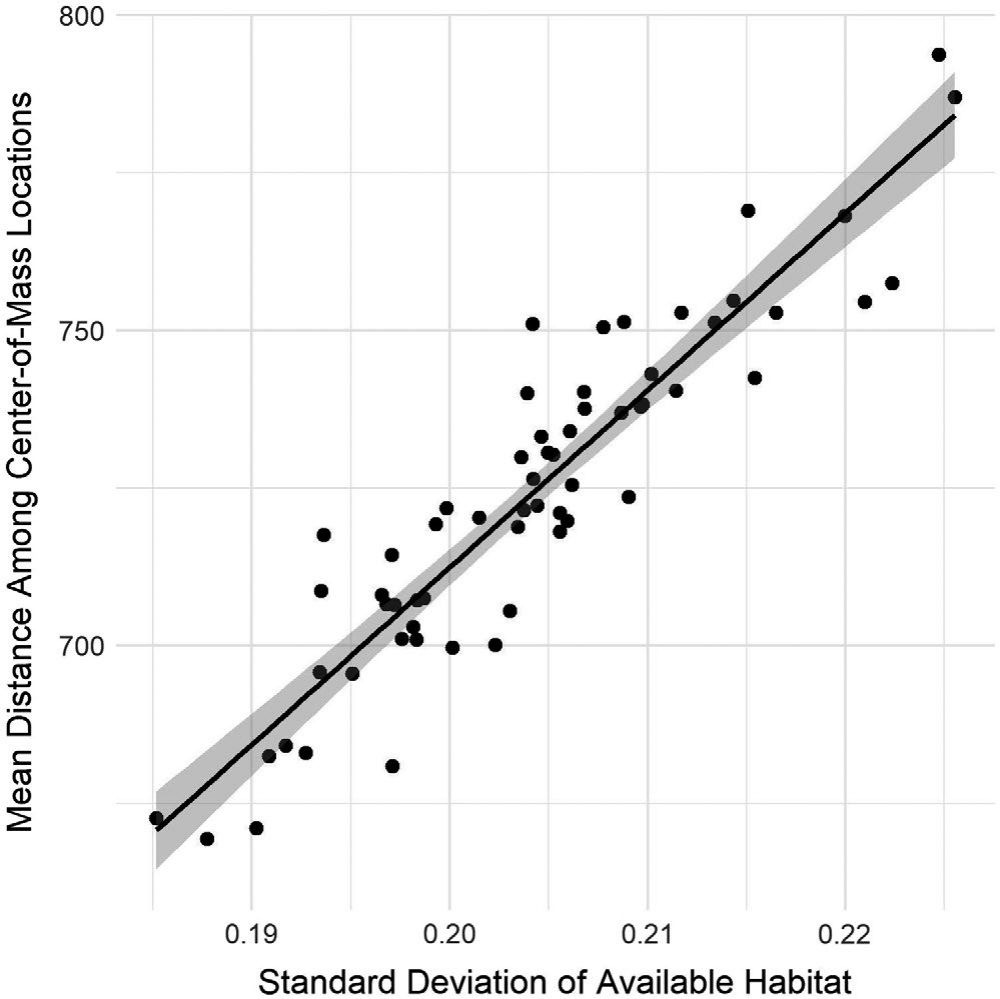
The mean distance among all abundance‐weighted center‐of‐mass locations for the population on each day increased as the standard deviation of the daily proportion of available habitat (weather severity index < 7.5) increased

We were successful in our attempt to recover the primary avenues for migration (Figure 7 and File S4): a heavily used central flyway along the Mississippi River, a well‐defined Atlantic Flyway east of the Appalachian Mountains, and a disjointed Pacific Flyway along the west coast. The flyways tended to converge within the Prairie Potholes Region and along the southern shore of Hudson Bay (which is the NatureServe defined breeding region). The most commonly used nodes during the course of the non‐breeding period were generally consistent across years (Figure 7). The center‐of‐mass of the population during the migratory periods were similarly consistent across years, while the overwintering period showed more inter‐annual variation (Figure 7). Finally, the model yielded strong evidence of the effect of *WSI* on the distribution of mallards on the landscape. The mean distance among all daily center‐of‐mass locations was highly correlated with the standard deviation of available habitat (adj.‐*R* ^2^ = 0.86; Figure 8) indicating that the distribution of mallards on the landscape changed more dramatically when the variation in daily available habitat was greater.

**FIGURE 9:**
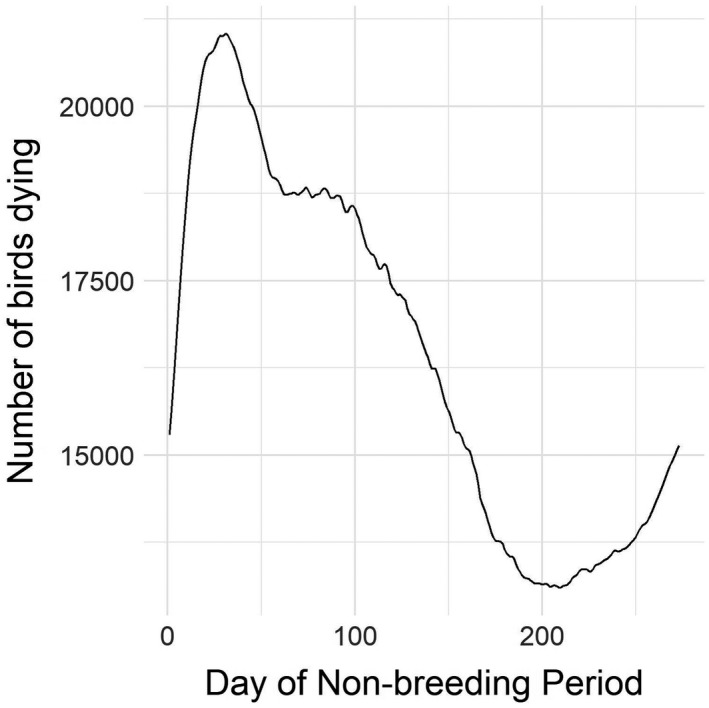
Mean daily mortality (total number of dead birds) on each day of the non‐breading period across the period of record (1957–2019)

Temporally, we were able to distinguish between severe and mild years by their mean daily WSI values across the landscape, and the years identified as severe or mild cohered to historical weather anecdotes. We found that years with severe weather yielded correspondingly reduced available habitat during the winter months (Figure 9). Finally, we found that as WSI increased, the population moved farther south during the non‐breeding period as available habitat was reduced (Figure 9, and see Appendix S3).

### 3.2 Validation

**FIGURE 10:**
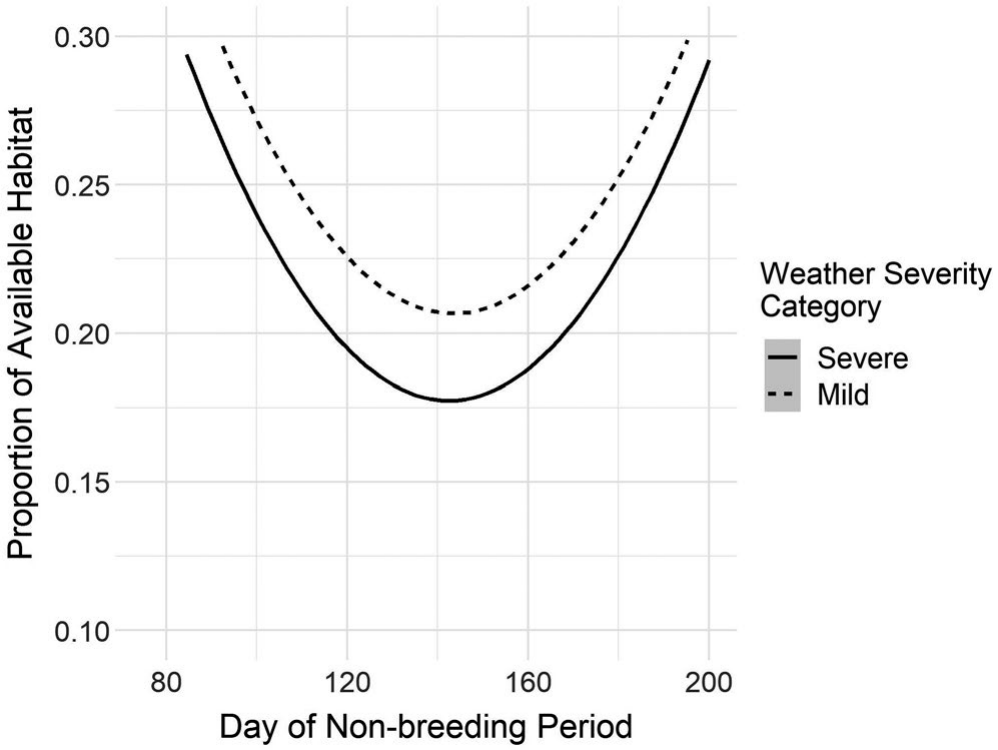
Comparison of mean daily proportion of available habitat during the non‐breeding period for years in the highest quartile of mean annual weather severity index values (“Severe”; 1961, 1964, 1966, 1971, 1972, 1974, 1975, 1977, 1978, 1981, 1982, 1984, 1993, 1995, 1996, and 2013) and for years in the lower quartile (“Mild”; 1979, 1980, 1997, 1998, 1999, 2001, 2003, 2004, 2005, 2006, 2009, 2011, 2015, 2016, and 2017)

Our model predicted an average survivorship of 77.4% for the non‐breeding period across all years (range = 76.4–78.4%). This value is decomposed into an average survivorship rate of 91.3% for the autumn migratory period (1 September to 30 November; 90.5–92.1%), 91.8% for the overwintering period (1 December to 28 February; 91.1–92.6%), and 92.4% for the spring migratory period (1 March to 31 May; 91.5–92.9%). Mean daily mortality across the period of record ranged from 13,100 to 21,000 mallards (Figure 10).

### 3.3 Parameter variation

The proportion of mallards in different body condition bins varied most strongly at lower classes, with a two‐order of magnitude increase in the proportion of mallards in the lowest body condition (starvation) over the non‐breeding period, and a 20% reduction in mallards in the top body condition. Decreasing the proportion of metabolizable body fat to 8% resulted in survivorship (averaged across all years) of 86.72% (85.87–87.37%), whereas increasing it to 14% metabolizable body fat yielded survivorship of 50.66% (50.00–51.35%). Increasing the coefficient for the fuel deposition rate to 2 increased survivorship to 89.15% (89.05–89.22%), whereas decreasing it to 0.5 decreased survivorship to 41.03% (39.99–41.95%) (while holding the proportion of metabolizable body fat steady at 11%).

## 4 DISCUSSION

We elaborated an energetics‐based model of avian migration to more fully realize the stochastic variation in migration induced by daily weather. Our model was able to recreate documented North American avian migration routes (La Sorte, Fink, Hochachka, DeLong, et al., 2014; La Sorte, Fink, Hochachka, Farnsworth, et al., 2014; Lonsdorf et al., 2016) and recover expected rates of survivorship (Lonsdorf et al., 2016) based on nothing more than first‐principle arrangements of dabbling duck energetics and behavior. These estimates are commensurate with literature‐derived mortality estimates (Davis et al., 2011; Zimmer et al., 2010). With a thorough literature review and carefully considered parameterization, the model we present here can be generalized to any migratory bird species. Extending the model to the entirety of the non‐breeding period is a crucial step on the path to developing a generalizable energetics‐based full‐annual‐cycle model (Marra et al., 2015). We included consideration of weather severity on the movement patterns of migrants, allowing us to form initial expectations about the role climate and climate change can play in altering physiology and subsequent migration behavior (Notaro et al., 2016). We introduced a refined forage availability scheme by allowing for consumption and natural decay of forage material during the non‐breeding period.

### 4.1 Interpreting results

Our model indicates that the milder conditions across North America resulting from climate change (Appendix S3; Schummer et al., 2017) are increasing the proportion of habitat available to wintering dabbling ducks. Our model revealed a concurrent decrease in mortality. This result is evident in the decrease in WSI over time across the continent, demonstrating generally less severe winters over the period of record. This result is also evidenced in the relationship between WSI and the minimum proportion of available habitat, with less available habitat in years with greater WSI.

Walther et al. (2002) indicated that freeze‐free periods were lengthening and that snow cover has decreased since the 1960s. If these trends continue, as recent studies suggest (Notaro et al., 2014), we may expect to see more northerly overwintering (Abraham et al., 2005; Link et al., 2006; Notaro et al., 2016; Tingley et al., 2009). Taken to the extreme, this development may indicate that mallard‐like dabbling ducks could be approaching a cessation of migration (Aagaard et al., 2018; Moore, 2011; Notaro et al., 2016). Recent studies investigating the changing patterns of avian migration under the influence of climate change provide corroborating evidence of this possibility (La Sorte & Thompson, 2007; Walther et al., 2002); American black ducks (*Anas rubripes*), for instance, have shown a tendency to remain in the region in which they breed during migration, and some occasionally move in directions antithetical to conventional migratory movements (Brook et al., 2009; Robinson et al., 2016). Whether this movement represents inexperienced birds or the influence of climate or land use/land cover change has not been decisively determined, but mounting evidence of similar patterns paired with the findings of this and other simulation models (Aagaard et al., 2018) indicate it is the latter.

By varying the proportion of available metabolizable body fat we were able to identify sensitivity within the model. The effect of modifying the proportion of available metabolizable body fat was counterintuitive; increasing body fat functionally increases available fuel and is expected to decrease time spent migrating, the most energy‐expensive aspect of the non‐breeding period. However, the proportion of body fat does not influence the cost of flight, so two individuals of the same body mass but different body fat proportions will be subjected to the same energetic costs. The individual with a greater proportion of body fat will be able to travel farther, decreasing its body mass more substantially and (based on the structure of our model) subsequently transitioning into a lower body condition class with a lower associated daily survivorship. If these aspects are correct, there likely exists an optimal arrangement of physiology (metabolizable fat) and behavior (flight distance) maximizing survival (Alerstam & Hedenström, 1998; Alerstam & Lindström, 1990), one that may change in concert with climate conditions.

Given the harsher conditions and limited habitat availability during the overwintering period (see Appendix S3), the lower survivorship is expected. In autumn, we expect greater forage availability on the landscape than in winter (and possibly even spring), as seed and waste grain has not yet decayed (Hagy & Kaminski, 2012), so we expected higher survivorship during this period. However, the timing of energy expensive migration fell in the autumn period (1 September to 30 November), which led to greater reductions in body condition and therefore generally greater mortality rates. The higher mean survivorship rate of spring is likely a result of less intense weather severity than in either autumn or non‐breeding periods.

When reviewing extreme weather events within the period of record we considered, there is appreciable concordance between WSI and observed extremes (e.g., deep freezes in the south, as in 1957–1958 and 1961–1962). Events such as these, coupled with our model results, offer support for the claim that poor weather tends to push birds farther south in search of hospitable habitat (Figure 5, Pavón‐Jordán et al., 2019). Conversely, mild years (such as 2015–2017) provide more available habitat across the landscape (Figures 9 and 10), likely leading to the population generally staying closer to the breeding grounds and demonstrating more willingness to withstand brief inclement weather, with the expectation that more hospitable conditions await after it quickly passes. As climatic conditions increase in variability this change could have dramatic effects on migratory dynamics, as some years may see mallards move only a short distance from the breeding grounds, while in other years, composed of extreme weather events, mallards may be pushed relatively far south. If the tendency of waterfowl is to remain sedentary as extreme events pass through, and if these events end up lasting longer, this sedentary inclination could lead to unusually high mortality events in some years (Newton, 2006, 2007). Historical data show a clear divergence in the spatial variation in weather severity, consistent with expectations of increasingly extreme weather as the climate changes, so predictions of general trends will necessarily be obscured by these spatially inconsistent weather pattern changes.

Waterfowl enthusiasts (e.g., birders and hunters, Cooper et al., 2015) contribute >$100 million annually to the economies of Canada and the United States (Mattsson et al., 2018, 2020). Should migration distance continue to shorten and sedentary behavior increase, the availability of waterfowl to birders and hunters would likely be affected, potentially leading to decreased funding in support of wetland habitat conservation (Cooper et al., 2015; Grado et al., 2001; López‐Hoffman et al., 2017). If hunter behavior were to change in response to differing migratory patterns, the distribution of monetary resources would likely change as well (Bagstad et al., 2018; López‐Hoffman et al., 2017; Mattsson et al., 2020).

### 4.2 Potential future research directions

Our model has many important strengths in terms of advancing our understanding of avian non‐breeding movement patterns within the context of energetics and weather. We sought to maintain flexibility in the model for ease of adding components that might increase the power of the model. We did not add these components in the present iteration because, in some cases, there remain critical gaps in our knowledge. For example, while we included a placeholder for harvest‐induced mortality, an important aspect of migration dynamics (Klaassen et al., 2005; Vaananen, 2001), we lacked access to data at the relevant spatial scale to inform the effect of this source of mortality across the landscape. Efforts to aggregate such data for inclusion in future iterations of this model would be useful. Because waterfowl migration is mediated on a daily time‐step via weather, forecasting waterfowl availability on time horizons useful to hunters could be possible.

While we used pertinent land cover data to inform forage availability across the landscape, we are aware of limitations in converting land cover classes into available kilojoules, as well as grouping potentially distinct land cover classes into broad categories (Malishev & Kramer‐Schadt, 2021). Targeted research into the seasonally varying availability of accessible forage (including invertebrates) in various land cover classes is necessary to better inform this aspect of the model (Beatty et al., 2017; Bishop & Vrtiska, 2008; Fredrickson & Reid, 1988; Kaminski et al., 2003). Understanding how caloric availability changes over the season, among years, and across space at resolutions coincident with the model can increase realism. Improving the reliability of spatial data layers is of particular importance to eIBMs, given the significance of this input on the resulting dynamics predicted by the model (Malishev & Kramer‐Schadt, 2021). Similarly, despite formatting our model with an agent‐based framework rather than individual‐based framework, we ignored another main challenge eIBMs face (Malishev & Kramer‐Schadt, 2021): accounting for complex behavior and movement (e.g., sociality and predation avoidance). Refining our understanding of the probabilistic tendencies of individuals to alter movement dynamics as a function of social dynamics or predation threat would greatly improve our approximation of especially small‐scale (short‐distance) movement.

In other cases, we omitted potentially important components because the complexity they add to the model significantly inflates computational time. We foresee a framework for adding in additional components in a serial, stepwise process. That is, we first developed a generalizable energetics‐based landscape model for avian migration (Lonsdorf et al., 2016), then laid the foundation for the interaction between temperature and migration energetics (Aagaard et al., 2018), and now merge those efforts to generate a generalizable continental‐scale energetics‐based landscape model of avian migration accounting for variable temperature and weather severity and their effects on migratory dynamics. By building toward the ultimate goal of a fully generalizable and energetics‐based animal movement model one block at a time, we provide a cogent workflow and fully elaborate the logic at each step. Thus, we have for now ignored the effect of some aspects such as wind direction on avian migration dynamics, a component known to be predictive of movement patterns (La Sorte, Fink, Hochachka, Farnsworth, et al., 2014). Adding model functions and data relating to daily wind currents and velocity would likely improve the realism of our model and provide refined predictions for migration routes and critical habitat areas (Gutierrez Illan et al., 2017).

We also excluded competition (Eichhorn et al., 2009; Stirnemann et al., 2012) and epidemiological effects (Gilbert et al., 2006) from the model. While we found that *WSI* has generally increased over time and led to decreased mortality, it is possible that altered disease dynamics may counteract these gains in survivorship (i.e., as the climate becomes milder, disease transmission may increase; Harvell et al., 2002), while decreased competition may provide the opposite influence. Developing techniques to account for these dynamics in the model would be beneficial. As with all models, the desired realism in the model needs to be balanced against the increased complexity necessary to deliver that realism.

Lastly, we made preliminary connections between the body condition of mallards at the end of the non‐breeding period and the energy available for reproduction during the breeding period. Assuming an energy conversion of 39,700 kJ per kg of body fat (Rayner, 1990), and an energy content of 400–636 kJ per egg (636 kJ in Ricklefs, 1977; 487 kJ in Sotherland & Rahn, 1987; 400 kJ in Alisauskas & Ankney, 1992), we estimate that, for 400 kJ per egg, only mallards with body mass >0.725 kg would have sufficient fat reserves available at the outset of the breeding period to lay at least one egg, and for 636 kJ per egg, only mallards with body mass >0.8 kg would be able to lay at least one egg (see Krapu, 1981 for discussion of body condition and breeding period success). Given a clutch size range for mallards of 8–13 eggs (Drilling et al., 2020), we estimate that only mallards with body mass >1.4 kg (for 400 kJ per egg) or >1.625 kg (636 kJ egg) would lay a full clutch of eggs at the beginning of the breeding period. Mallards in lower body conditions would need more time to forage to sufficiently restock fat reserves to produce a full clutch size. The simulated distribution of mallards in each body condition at the end of the non‐breeding period indicates that approximately 3% (for 636 kJ per egg) to 15% (for 400 kJ per egg) of the population could effectively lay a standard size clutch of eggs at the beginning of the breeding period, also allowing for the possibility of a second clutch (depending on the size of each) later in the breeding period given a rapid enough rate of fuel deposition.

### 4.3 Conclusions

Accelerating climate change is affecting avian migration. Merging environmental conditions with spatially explicit models of energetics‐based migratory movements is helping to inform how the landscape affects migration patterns. Our model approximates avian migration during the non‐breeding period and the movement occurring among local stopovers along the way. Our results indicate that hospitable areas during the non‐breeding period have increased over time, indicative of milder conditions as a product of a changing climate, ultimately leading to decreased (environmentally induced) mortality. This finding has important ramifications: if migration distance continues to diminish and the tendency for sedentary behavior increases, we may see altered hunter harvest across the landscape. Additionally, if sedentary behavior in the face of extreme events continues then mallards may experience unusually high mortality events in some years. All these possibilities underscore the benefit of continued advancements in the vein of this model to further illuminate the consequences of a changing environment on avian migration.

## CONFLICT OF INTEREST

The authors report no conflicts of interest.

## AUTHOR CONTRIBUTIONS

**Kevin J. Aagaard:** Conceptualization (equal); Data curation (lead); Formal analysis (lead); Methodology (equal); Validation (lead); Visualization (lead); Writing – original draft (lead). **Eric V. Lonsdorf:** Conceptualization (equal); Writing – review & editing (supporting). **Wayne E. Thogmartin:** Conceptualization (equal); Formal analysis (supporting); Funding acquisition (lead); Methodology (supporting); Supervision (equal); Validation (supporting); Visualization (supporting); Writing – review & editing (equal).

## Supporting information

Appendix S1

## Data Availability

All data are stored in publicly available repositories as cited in the paper (e.g., weather data comes from the National Oceanic and Atmospheric Administration's National Centers for Environmental Prediction). We provide all code as Supporting Information, and the code has annotated references to each dataset.
