#  Corrigendum

**DOI:** 10.1002/ece3.9148

**Published:** 2022-07-20

In the recent article by Aagaard et al. (2022), the out‐of‐date USGS statement has been removed in the Acknowledgments of the article and it now reads as follows:
