# Supplementary material for: Effects of weather variation on waterfowl migration: Lessons from a continental‐scale generalizable avian movement and energetics model
Source: Ecol Evol. 2022 Feb 17;12(2):e8617. doi: 10.1002/ece3.8617 (PMC8853969; doi:10.1002/ece3.8617)
Supplement: Supplementary file 1 — Appendix S1 [file ECE3-12-e8617-s001.zip › ece38617-sup-0002-Methods.pdf]

Details on the methods used in Lonsdorf et al. (2016) to generate the energetic landscape across which we propel our simulated bird population.

The energetic landscape was derived in Lonsdorf et al. (2016). We wrote then that the abundance of forage material on the landscape is influential in determining the number of birds that can use a site during a migratory period. We used the following equation to estimate the forage availability of a stopover site (Lonsdorf et al. 2016, equation 1.2):

$$HF_j = A_j \sum_{c=1}^{C} K_c p_{cj}, \qquad (1.2)$$

With equation 1.2 we assumed that the proportion, p_{cj}, of a site, j, covered by land cover, c, and the amount of forage, K_c (in kiloJoules), provided by a particular land cover class (per unit area of the site, A_j). This equation therefore produced the potential amount of forage provided in a site, HF_j.

We then estimated available food energy per land cover pixel using equation 1.2.1 form Lonsdorf et al. (2016):

$$\bar{H}\bar{F}_j = A_j \sum_{c=1}^{C} K_c p_{cj} q_{cj}, \qquad (1.2.1)$$

Here, q_{cj} is equal to the proportion of forage available based on distance to the nearest roost site (details of how we determined q_{cj} can be found in Lonsdorf et al. [2016], Appendix S4). Combining the quantity of forage for fall (Lonsdorf et al. [2016], Fig. 1E) and spring (Lonsdorf et al. [2016], Fig. 1F) periods relative to roosting habitat provides overall assessment of habitat quality for fall and spring migration.

We informed parameters for equation 1.2.1 using literature-derived values pertaining to Mallard food habits (Lonsdorf et al. 2016, Appendix S3). The proportion of forage within a site that is available to a Mallard is represented as a function of a forage location's distance to the nearest roosting site. Because of increasing travel costs as the distance between a stopover site and the nearest roosting site increases, the net energy gained, which is the realized quality of a forage site, will decline (Johnson et al. 2014).

References

Johnson, W. P., P. M. Schmidt, and D. P. Taylor. 2014. Foraging flight distances of wintering ducks and geese: a review. Avian Conservation and Ecology 9:2.

Lonsdorf, E. V., Thogmartin, W. E., Jacobi, S., Aagaard, K., Coppen, J., Davis, A., Fox T., Heglund P., Johnson R., Jones T., Kenow K., Lyons J., Luke K., Still S., & Tavernia, B. (2016). A generalizable energetics-based model of avian migration to facilitate continental-scale waterbird conservation. *Ecological Applications* 26:1136–1153.
