# Supplementary material for: Effects of weather variation on waterfowl migration: Lessons from a continental‐scale generalizable avian movement and energetics model
Source: Ecol Evol. 2022 Feb 17;12(2):e8617. doi: 10.1002/ece3.8617 (PMC8853969; doi:10.1002/ece3.8617)
Supplement: Supplementary file 1 — Appendix S1 [file ECE3-12-e8617-s001.zip › ece38617-sup_0001-Supinfo.docx]

**Supporting Material**

We provide several files of supporting material for visualization of our results and replication of our methods.

**File S1**. (game_distrib.R) – R script used to apply the model and generate the results.

**File S2**. (Details on Lonsdorf et al. 2016 methods.pdf) – Summary documentation of the methods used in Lonsdorf et al. 2016 relevant to the present study.

**File S3**. (weather_severity_index.R) – R script used to acquire the data and perform the calculations to generate the weather severity index for each day of the simulations.

**File S4**. (DailyAbundance_2013.gif) – A video showing the daily abundance per node across the landscape, normalized on a zero-to-one scale for 2013. White to gray dots depict the population-weighted center of mass on each day of migration to demonstrate an estimate of the population-level “migration path.”

**Appendix S1. Details of the cleaning, conversion, and projection of the weather data from the National Oceanic and Atmospheric Administration’s National Centers for Environmental Prediction.**

We used the RNCEP package (R-connection to National Centers for Environmental Prediction data; Kemp et al. 2012) to gather and manipulate the target climate data, which included data for each day between 1957 and 2019. We gathered air temperature (°K at 2 m above surface level), water equivalent of snow depth ($\frac{kg}{m^{2}}$ at surface level), and air pressure (Pascals at low cloud bottom) data sampled on a T62 Gaussian grid. We restricted data to the non-breeding period, from 1 July of one calendar year to 31 May of the next (ignoring leap days). We converted air temperature to °C and water equivalent of snow depth to meters. To convert air pressure to density ($\frac{kg}{m^{3}}$), we divided the pressure by the product of the specific gas constant for dry air (287.058 $\frac{J}{kg*km}$) and air temperature.

The water equivalent of snow depth (or, snow-water equivalent, SWE) measures the amount of water that would be released by a volume of snow melting. It is calculable as the product of snow depth and snow density. To acquire snow depth (in m) given SWE, we took the quotient of SWE ($\frac{kg}{m^{2}}$) and snow density ($\frac{kg}{m^{3}}$). Snow density varies with temperature, and pressure (or depth, with snow deeper in a column being more compacted and thus denser). Snow density ranges from 10 to 400 $\frac{kg}{m^{3}}$ in our conditions (i.e., the temperatures observed across the focal landscape); we assume a constant snow density of 400 $\frac{kg}{m^{3}}$ across the landscape to convert SWE to snow depth.

**References**

Kemp, M. U., E. Emiel van Loon, J. Shamoun-Baranes, and W. Bouten. 2012. RNCEP: global weather and climate data at your fingertips. Methods in Ecology and Evolution 3:65–70.

**Appendix S2.** **Cross-walk of Canada (CSC2000v, Center for Topographic Information, Earth Sciences Sector and Natural Resources Canada 2009) and U.S. (National Land Cover Database 2006, Fry et al. 2011) land cover.**

| CSC Value | CSC Class | NLCD Value | NLCD Class |
| --- | --- | --- | --- |
| 0 | No Data | No Data |  |
| 10 | Unclassified | No Data |  |
| 11 | Cloud | No Data |  |
| 12 | Shadow | No Data |  |
| 20 | Water | 11 | Open Water |
| 30 | Barren | 31 | Barren Land |
| 31 | Snow/Ice | 12 | Perennial Ice/Snow |
| 32 | Rock/Rubble | 31 | Barren Land |
| 33 | Exposed Land | 31 | Barren Land |
| 34 | Developed | 23 | Developed, Medium Intensity |
| 35 | Sparsely vegetated bedrock | 31 | Barren Land |
| 36 | Sparsely vegetated till-colluvium | 31 | Barren Land |
| 37 | Bare soil with cryptogam crust - frostboils | 31 | Barren Land |
| 40 | Bryoids | 74 | Moss |
| 50 | Shrubland | 52 | Shrub/Scrub |
| 51 | Shrub -Tall | 52 | Shrub/Scrub |
| 52 | Shrub - Low | 51 | Dwarf Scrub |
| 53 | Prostrate dwarf shrub | 51 | Dwarf Scrub |
| 80 | Wetland | 95 | Emergent Herbaceous Wetlands |
| 81 | Wetland Treed | 90 | Wood Wetlands |
| 82 | Wetland Shrub | 90 | Wood Wetlands |
| 83 | Wetland Herb | 95 | Emergent Herbaceous Wetlands |
| 100 | Herb | 71 | Grassland/Herbaceous |
| 101 | Tussock graminoid tundra | 72 | Sedge/Herbaceous |
| 102 | Wet sedge | 72 | Sedge/Herbaceous |
| 103 | Moist to dry non-tussock graminoid/dwarf shrub tussock | 51 | Dwarf Scrub |
| 104 | Dry graminoid prostrate dwarf shrub tundra | 51 | Dwarf Scrub |
| 110 | Grassland | 71 | Grassland/Herbaceous |
| 120 | Cultivated agricultural land | 82 | Cultivated Crops |
| 121 | Annual crops | 82 | Cultivated Crops |
| 122 | Perennial crops and Pasture | 81 | Pasture/Hay |
| 210 | Coniferous | 42 | Evergreen Forest |
| 211 | Coniferous - Dense | 42 | Evergreen Forest |
| 212 | Coniferous - Open | 42 | Evergreen Forest |
| 213 | Coniferous - Sparse | 42 | Evergreen Forest |
| 220 | Broad Leaf | 41 | Deciduous Forest |
| 221 | Broad Leaf - Dense | 41 | Deciduous Forest |
| 222 | Broad Leaf - Open | 41 | Deciduous Forest |
| 223 | Broad Leaf - Sparse | 41 | Deciduous Forest |
| 230 | Mixed Wood | 43 | Mixed Forest |
| 231 | Mixed Wood - Dense | 43 | Mixed Forest |
| 232 | Mixed Wood - Open | 43 | Mixed Forest |
| 233 | Mixed Wood - Sparse | 43 | Mixed Forest |

**References**

Center for Topographic Information, Earth Sciences Sector and Natural Resources Canada. 2009. Land cover, circa 2000-vector: Feature catalogue, edition 1.0. Geobase, http://www.geobase.ca/geobase/en/data/landcover/csc2000v/description.html.

Fry, J., G. Xian, S. Jin, J. Dewitz, C. Homer, L. Yang, C. Barnes, N. Herold, and J. Wickham. 2011. Completion of the 2006 National Land Cover Database for the Conterminous United States. Photogrammetric Engineering and Remote Sensing 77:858–864.

**Appendix S3. Here we report several results and findings relevant to the change in weather severity over time and the subsequent effects of this change on the nadir of the latitudinal reach of the population each year.**

**Figure S3.1.** The proportion of the landscape available for individuals to occupy on each day of the non-breeding period, according to the weather severity index (*WSI*) in that node. *WSI* values above 7.5 trigger departure and inhibit arrival. This metric is normalized to a 0 to 1 scale for comparison across all years in the record (1957 – 2019).

**
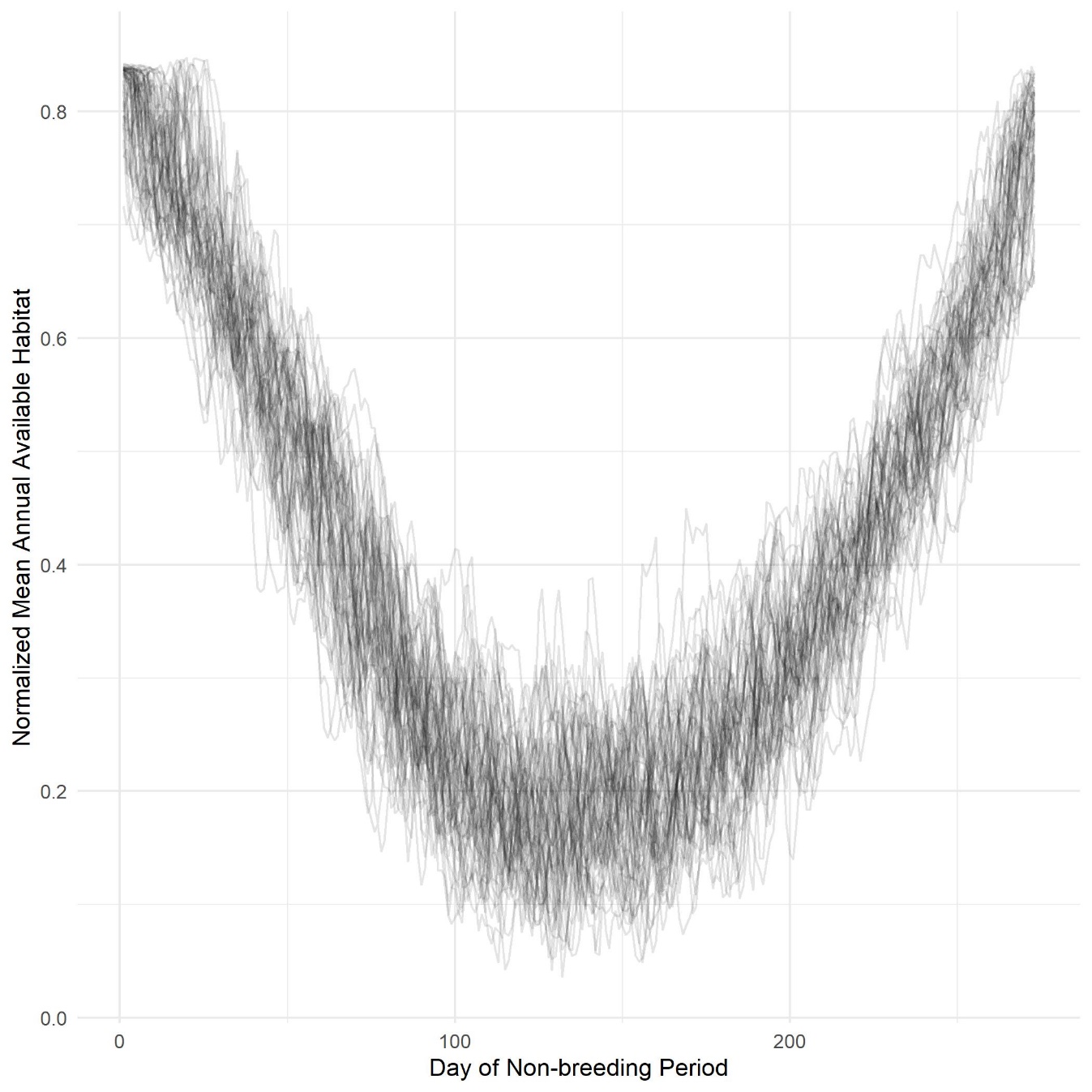
**

**Figure S3.2.** Change in the southernmost latitude achieved by the abundance-weighted center-of-mass of the population over time, with corresponding annual median weather severity index. Years of less severe weather (lighter curves) demonstrated less southerly minimum latitudes.

**
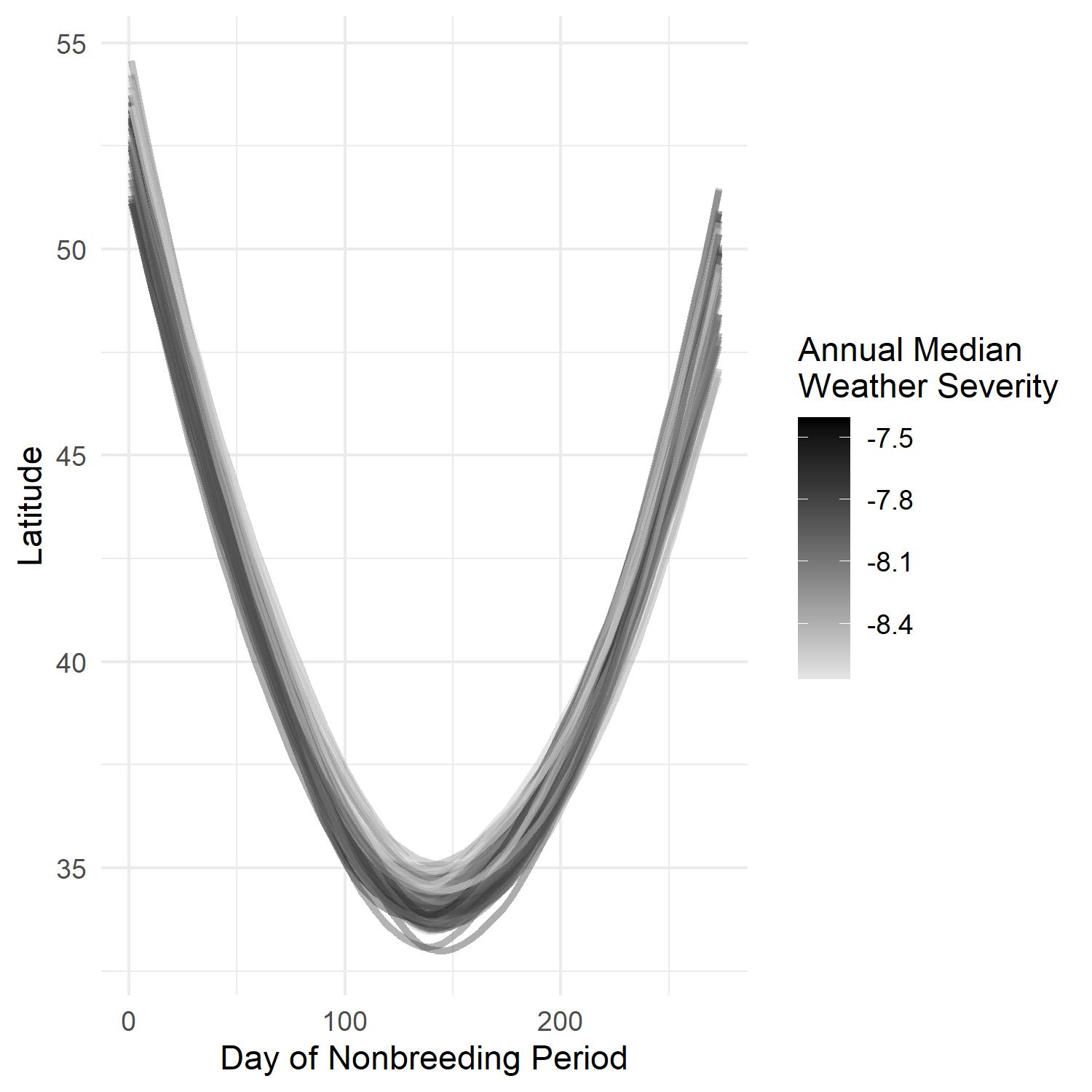
**

**Figure S3.3.** Change in the southernmost latitude achieved by the abundance-weighted center-of-mass of the population with varying annual median weather severity index values.

**
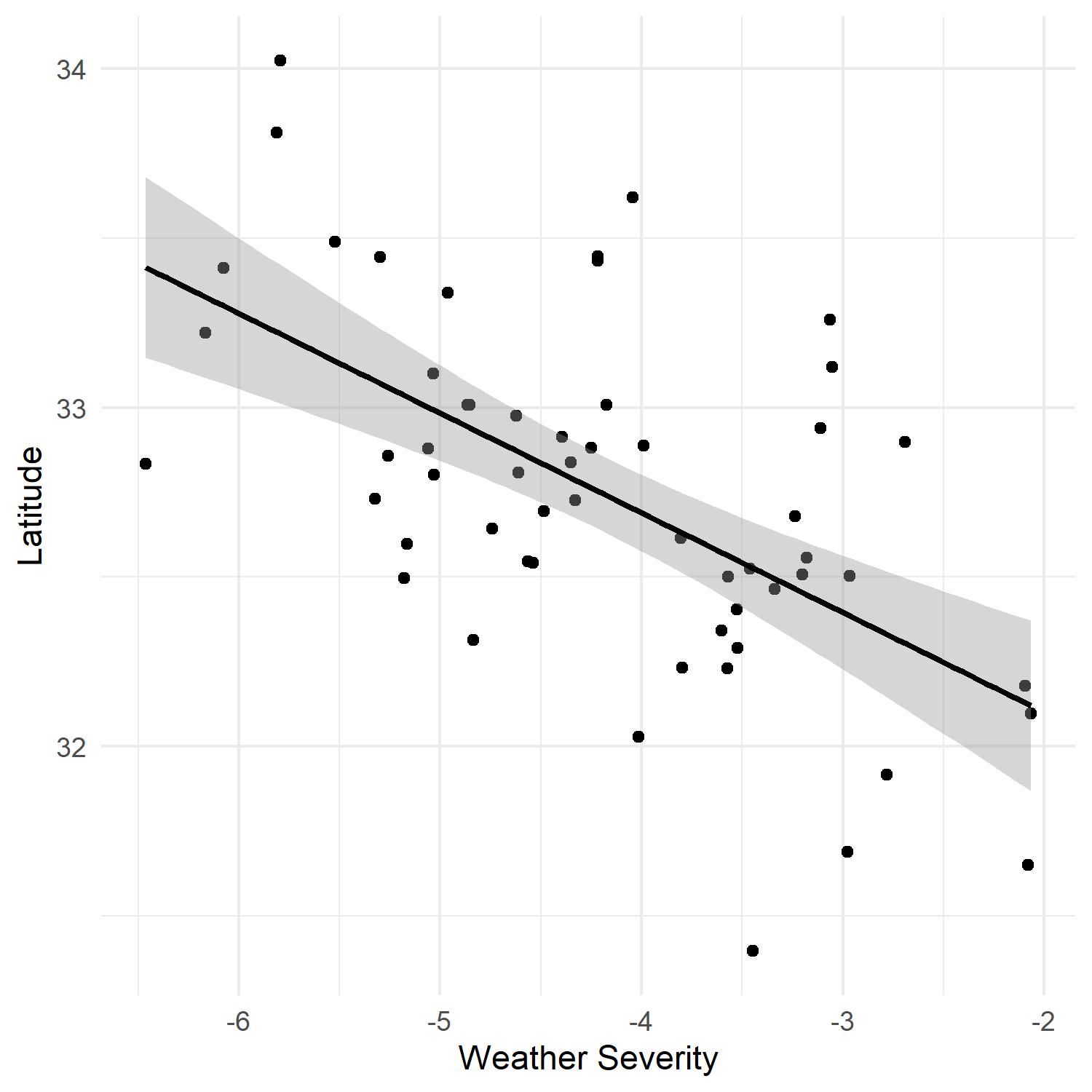
**
