# Supplementary figures and images for: Effects of weather variation on waterfowl migration: Lessons from a continental‐scale generalizable avian movement and energetics model
Source: Ecol Evol. 2022 Feb 17;12(2):e8617. doi: 10.1002/ece3.8617 (PMC8853969; doi:10.1002/ece3.8617)

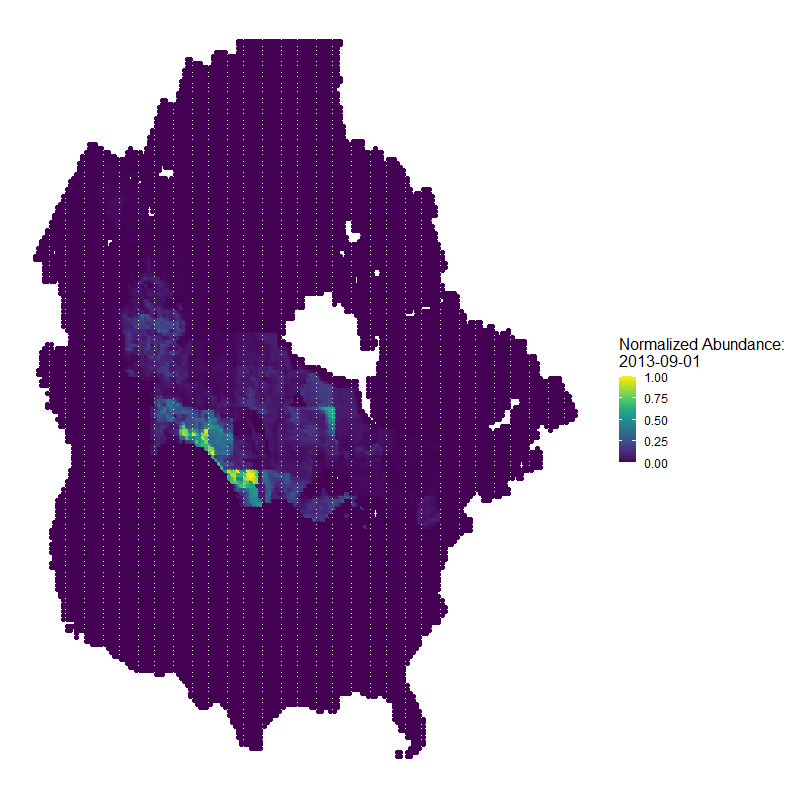

Supplement: Supplementary file 1 — Appendix S1 [file ECE3-12-e8617-s001.zip › ece38617-sup-0005-Supinfo.gif]
